# The Mini Colon Model: a benchtop multi-bioreactor system to investigate the gut microbiome

**DOI:** 10.1080/19490976.2022.2096993

**Published:** 2022-07-17

**Authors:** Zijie Jin, Andy Ng, Corinne F. Maurice, David Juncker

**Affiliations:** a Department of Biomedical Engineering, McGill University, Montreal, Quebec Canada; b McGill Genome Centre, McGill University, Montreal, QC, Canada; c Department of Microbiology and Immunology, McGill University, Montreal, QC Canada

**Keywords:** Microbiota, gut model, 16S rRNA gene, bioreactor, microbiome

## Abstract

*In vitro* fermentation systems allow for the investigation of gut microbial communities with precise control of various physiological parameters while decoupling confounding factors from the human host. Current systems, such as the SHIME and Robogut, are large in footprint, lack multiplexing, and have low experimental throughput. Alternatives which address these shortcomings, such as the Mini Bioreactor Array system, are often reliant on expensive specialized equipment, which hinders wide replication across labs. Here, we present the Mini Colon Model (MiCoMo), a low-cost, benchtop multi-bioreactor system that simulates the human colon environment with physiologically relevant conditions. The device consists of triplicate bioreactors working independently of an anaerobic chamber and equipped with automated pH, temperature, and fluidic control. We conducted 14-d experiments and found that MiCoMo was able to support a stable complex microbiota community with a Shannon Index of 3.17 ± 0.65, from individual fecal samples after only 3–5 d of inoculation. MiCoMo also retained inter-sample microbial differences by developing closely related communities unique to each donor, while maintaining both minimal variations between replicate reactors (average Bray-Curtis similarity 0.72 ± 0.13) andday-to-day variations (average Bray-Curtis similarity 0.81±0.10) after this short stabilization period. Together, these results establish MiCoMo as an accessible system for studying gut microbial communities with high throughput and multiplexing capabilities.

## Introduction

The gut microbiota has been increasingly recognized in its role for human health and disease.^1^ The trillions of microorganisms residing in the human gut respond to environmental factors such as diet and compounds foreign to the human body (xenobiotics), and have been found to profoundly impact human mental and physical condition, in addition to modulating disease progression and drug metabolism.^2^ The gut microbiota is characterized by high levels of inter- and intra-individual differences.^3–5^ As such, the study of individualized responses of the gut microbiota to perturbations often proves difficult, and various experimental technologies have been developed to limit or control these variables.

For example, gnotobiotic mice and human-microbiota-associated (HMA) mice are powerful models to study the response of either a defined microbial community or a complex one directly transplanted from human fecal samples. Such *in vivo* approaches, integrated with host interactions and immune responses, offer highly physiologically relevant experimental conditions. However, these systems are also expensive to use, requiring specialized animal facilities, and are limited by the inherent biological variability and animal housing conditions.^6,7^ Further, the incorporation of host interactions can confound the specific response of the microbiota from that of the host.^8^

*In vitro* fermentation systems that model various sections of the human gastrointestinal (GI) tract, on the other hand, allow for the investigation of microbial communities with precise control of various physiological parameters, such as nutrient availability and pH levels, while decoupling interference from the human host.^9^ Various systems with a range of complexity have been developed and implemented in the rapidly growing field of gut microbiome research. The Simulator of Human Intestinal Microbial Ecosystem (SHIME) system,^10^ which is probably the most well-known example, consists of 5-stage chemostat reactors mimicking different sections of the human GI tract. Similar systems include the TNO models and Robogut, all equipped with automatic control for physiological conditions and which can be set up as single or multi-stage reactors.^11–13^ While validated with several types of samples, these *in vitro* systems are large in footprint with liter-sized reactors, thereby limiting multiplex capabilities and experimental throughput, as the reactors typically require a few weeks for microbiota stabilization.^9,10,14^ The footprint also means that labs using these systems need to be well-equipped with dedicated spaces for running them.

Several teams have taken another approach, attempting to miniaturize these bioreactors, notably the Mini Bioreactor Arrays (MBRA) and the Mipro systems.^15–17^ The MBRA can run 24 different experiments in parallel with a working volume of 15 ml and has been used to study the effect of various emulsifiers on gut microbial diversity and composition.^18^ However, this system still relies on an anaerobic chamber for anoxic conditions and temperature control. In addition, it lacks pH control and relies on expensive multi-channel pumps and multi-point stir plates for fluidic transfer and mixing. In contrast, Mipro operates in batch mode and relies on manual sampling and refilling of bacterial media. Excelling at multiplexing (96 different experiments can run in parallel), this system is more suitable for quick and large-scale initial screening within 24–48 hours instead of time series experiments. However, Mipro is not equipped with a mixing system and similar to the MBRA, it requires an anaerobic chamber for anoxic conditions.^17^

Considering the above, there is a need for *in vitro* systems that fill the gap between existing systems in terms of footprint, physiological parameters controlled, stabilization time prior to experimental time, cost, and circumventing the need for an anaerobic chamber. Here, we present the Mini Colon Model (MiCoMo), a low-cost, miniaturized multi-bioreactor system that simulates the human colon with the capacity to change culture conditions to match physiological conditions or specific experimental needs. Consisting of triplicate 30-ml working volume reactors, MiCoMo allows for automatic and user-adjustable control of physiological conditions such as pH, temperature, anoxia, and media feeding schedule. The system has a small footprint thanks to the small working volume and operates independently of an anaerobic chamber. Fabricated without specialized material or parts, MiCoMo uses common disposable labware that can be acquired easily. Thus, its costs can be limited to a fraction of the currently available systems. We validated MiCoMo’s performance by investigating the growth of strict anaerobes, before monitoring the development and stabilization of microbial communities obtained from fecal samples of several healthy unrelated volunteers. We find that MiCoMo allowed for fast stabilization of complex microbial communities (<5 d), while sustaining microbial diversity from individual donors over the course of 14 d-experiments. Given the low cost and ease of operation, we believe MiCoMo is a suitable and accessible tool to conduct individualized human gut microbiota studies.

## Results

### Design, fabrication and components of MiCoMo

**Figure 1.**
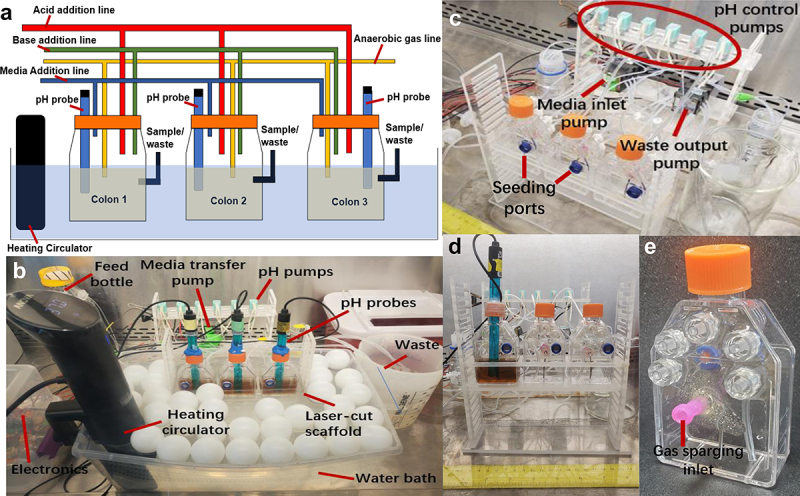
A) Schematic and B) operating photograph of MiCoMo showing major components. The reactors are kept in water bath during operation with ping-pong balls to minimize evaporation. The whole device is kept in BSC to minimize risks of contamination. C) Major pumps and tubing connection of MiCoMo. Two multi-channel pumps transfer media in and remove waste from all reactors, while two single channel pump control acid and base addition for each reactor through connection ports at the back of reactors. At beginning of each experiment fecal slurry is seeded into each reactor manually through seeding port at the front of reactor. D) Front view of MiCoMo, with one reactor with media and pH probe. E) Picture of individual MiCoMo reactor. Luer-lock ports connect media inlet, water removal, gas vent, gas sparge inlet and acid/base addition, respectively.

A schematic and images of MiCoMo are shown in Figure 1. MiCoMo consists of 3 single stage reactors with 55 mL capacity and 30 mL working volume. Each individual reactor is equipped with acid/base adjustment and fluidic transfer tubing with Luer-lock connectors, as well as a gas sparging line for N_2_ flushing to keep reactors anoxic. The N_2_ sparging also homogenizes the reactor contents and serves as the mixing system of MiCoMo. During the operation of MiCoMo, the anaerobic reactors, maintained at 37°C in a water bath, can be contained in a biosafety cabinet to avoid any potential contamination. MiCoMo operates on a 4-hour feed cycle for all experiments in this study. The feed cycle leads to an overall reactor turnover time of 30 hours.

### Validation of MiCoMo operations

**Figure 2.**
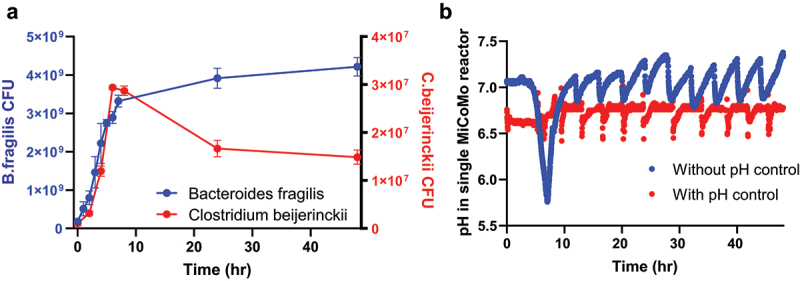
Validation of MiCoMo control system A) Growth curves of strict anaerobes in MiCoMo operating in batch mode B) Log of pH in MiCoMo seeded with fecal sample operating on automatic feeding cycle, with and without active pH control.

We first validated MiCoMo’s maintenance of anoxic condition with strict anaerobic bacterial isolates. We maintained pre-reduced PBS with 1 mg·L^−1^ resazurin in MiCoMo for 24 hours and confirmed no color change. Next, we grew the following strict anaerobes in the system: *Clostridium beijerinckii* (Gram +) and *Bacteroides fragilis* (Gram -). We observed the expansion of both strict anaerobes in MiCoMo reactors operating in batch mode. Within 48 hours’ experimental time, *B. fragilis* grew from 1.76 ***± ***0.61 × 10^8^ CFU·mL^−1^ to 4.32 ***± ***2.41 × 10^9^ CFU·mL^−1^ and *C. beijerinckii* grew from 1.10 **± **0.55 × 10^6^ CFU·mL^−1^ to 1.49 **± **0.15 × 10^7^ CFU·mL^−1^ (Figure 2a).

Next, we evaluated the effect of pH control on MiCoMo community dynamics by seeding MiCoMo with fecal samples from one volunteer and compared pH dynamics in the reactors with and without pH control (three technical repeats for each condition). We found that without pH control, the pH of MiCoMo quickly reduced from 7.0 to ~5.7 within the first 8 hours (2 feed cycles), suggesting a swiftly growing microbial community depleting initial nutrients. The pH then increased back to ~7.0 within the next 4–5 hours, possibly due to metabolic activities, such as metabolite build up and cell death. Interestingly, MiCoMo underwent cyclic pH fluctuations corresponding to the feed cycles from that point on, likely due to the same reasons as above. In contrast, with pH control, the pH of MiCoMo was kept at the setpoint pH of 6.7 with **±** 0.1 tolerance (values automatically adjusted within 10s); (Figure 2b)

### Stabilization of complex microbial communities derived from fecal samples

We inoculated MiCoMo with fecal samples from healthy volunteers to determine whether MiCoMo can sustain the growth of complex microbial communities. Four unrelated healthy volunteers were included in this study. A fifth sample was created by pooling fecal matter from two individuals at 1:1 weight ratio to explore the effects of pooling donor samples, a common approach for inoculating germ-free mice.^8,19,20^

**Figure 3.**
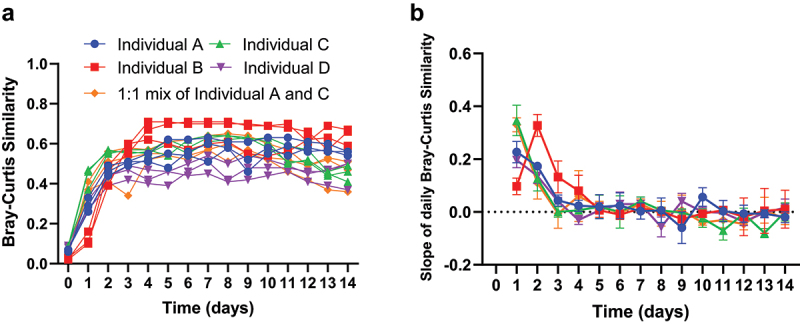
Stabilization of microbial communities derived from fecal samples in MiCoMo. A) Average Bray-Curtis similarity between each daily reactor sample and samples of all other days in that reactor. Day 0 indicates the original fecal sample. Each line indicates an individual reactor. B) Rate of change of Bray-Curtis similarity between consecutive days, evaluated by 3-point moving window average. Each line is average across 3 technical repeats of the same individual donor, error bars indicate standard deviations.

We examined the stability of microbial cultures based on a beta diversity metric, the Bray-Curtis similarity (1 – Bray-Curtis distance). We computed the Bray-Curtis similarity of each daily sample with the average of all other daily samples, termed averaged similarity thereafter, to gauge the long-term community stabilization (Figure 3a). Furthermore, we evaluated the daily Bray-Curtis similarity between consecutive days, termed daily similarity thereafter (Figure 3b), and computed its daily rate of change to evaluate short-term community dynamics (Supplementary Figure S4).

For all volunteers, except individual B, we found that microbial communities changed rapidly over the first 24 hours of inoculation, with averaged similarity quickly increasing from <0.1 to around 0.5. The rate of change of community structure quickly decreased after this initial transition period, but the overall community underwent another 24 h of transition before reaching a relatively stable state: we observed a rate of change of 0.28 **±** 0.07 during the 24-h transition period (Day 1) and 0.14 **±** 0.03 within the next 24 hours (Day 2). The community then transitioned to a stable state with rates of change less than **±** 0.025. During this period the averaged similarity reached a plateau between 0.45 and 0.55, depending on the individual, and a mean daily similarity of 0.80 **±** 0.09 (all volunteers average, except individual B).

Meanwhile, the fecal inoculum from individual B showed different stabilization patterns: the major transition period for individual B occurred on Day 2 instead of Day 1, with a rate of change of 0.33 **± **0.04 and 0.10 **±** 0.03, respectively, while the averaged similarity showed similar behavior as the other individuals. Further, the microbial community from individual B took additional time to reach stability with slopes of 0.13 **±** 0.01 and 0.08 **±** 0.06 on Day 3 and 4, respectively. Starting from Day 5, this community entered a stabilized state similar to that from the other volunteers, indicated by the plateauing of averaged similarity and characterized by a high daily similarity of 0.87 **±** 0.08 with minimal fluctuations for the rest of the 14-d period. Interestingly, following this longer transition period, the microbial community developed into a stable state with similar day-to-day variations, but overall higher averaged similarity, around 0.6, relative to the other individuals.

After analyzing the temporal stability of MiCoMo inoculated with complex microbial communities, we then compared the replicate reactors within each MiCoMo run from the same original donor to gauge the consistency of these technical replicates. We found that MiCoMo can maintain highly consistent technical replicates: the between-replicate similarity ranged from 0.41 to 0.9, with an average of 0.72 **±** 0.13 and a median of 0.74. We found that the mixed fecal matter inoculum has a lower between-replicate similarity (0.63 **±** 0.12) comparing to the other donors (0.74 **±** 0.13), although this difference is not statistically significant (p > .05, unpaired T-test). This could be due to the development of distinctive stochastic community structures in the replicate reactors.

### Diversity dynamics and structure of microbial communities in MiCoMo

Having established that MiCoMo can lead to stabilized microbial communities in 3–5 d, we examined the alpha diversity and structure of these communities to evaluate how representative they are relative to the original fecal inocula.

**Figure 4.**
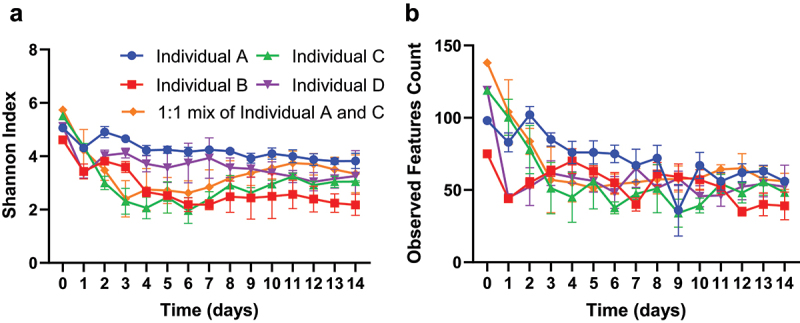
Alpha diversity of microbial communities derived from fecal samples in MiCoMo. A) Alpha diversity measured by Shannon Index. B) Observed features (ASVs) count. Each line is average across 3 technical repeats of the same individual donor, error bars indicate standard deviations.

Here, we adopted one commonly applied metric for alpha diversity, the Shannon index. We found that fecal samples included in this study have a Shannon index between 4.62 and 5.73, and that the transfer and growth of fecal samples in MiCoMo lead to a slight decrease in Shannon index for all individuals (Figure 4a). This decrease was most noticeable during the first 24 hours, with the average Shannon index significantly decreasing from 5.21 ± 0.43 to 3.13 ± 0.60 (p < .001, unpaired T-test). During the rest of the experimental period, the Shannon index in MiCoMo gradually fluctuated for a few additional days before stabilizing at 3.17 **±** 0.65 (average of all volunteers, Day 5–14). As expected, these transition patterns mirrored the similarity patterns discussed above. We also calculated the amount of observed ASVs within each MiCoMo sample (Figure 4b). We found the observed ASVs follow a similar trend as alpha diversity, and that stabilized ASV counts are typically ~50% of that observed from the original fecal sample.

**Figure 5.**
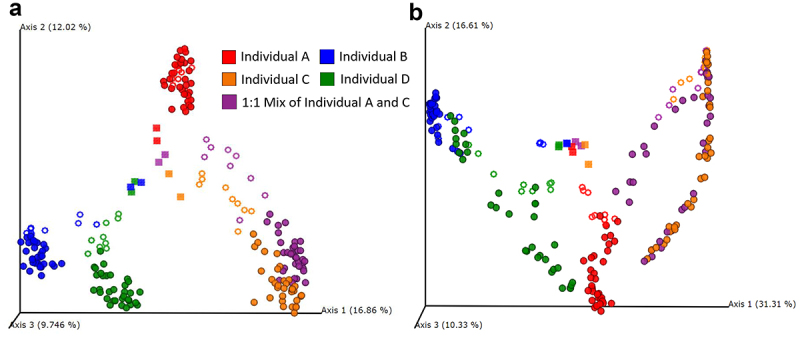
Principal component analysis on diversity and structure of microbial communities derived from fecal samples in MiCoMo by A) Jaccard Distance B) Bray-Curtis Distance. Color: samples from individual donors. Square: original fecal sample. Rings: samples from individual replicate reactors in Day 1–3. Circles: samples from individual replicate reactors in Day 4–14.

Next, we evaluated differences between MiCoMo communities and their respective original fecal sample. We computed the distance matrix between MiCoMo samples from different volunteers for two metrics, the Bray-Curtis similarity and Jaccard similarity (1 – Jaccard distance), which accounts only for the presence/absence of members within a community, as opposed to Bray-Curtis which also considers evenness. Acknowledging the fact that MiCoMo culture led to the loss of some bacterial taxa and an inevitable transition of microbial community structure, we were interested in whether communities from different individuals would develop and significantly cluster away from each other. To this end, we applied a permutational multivariate analysis of variance (PERMANOVA)^21^ on MiCoMo samples from each volunteer and analyzed the distance matrices by principal component analysis (Figure 5a, b). There is a clustering of the original fecal samples distinct from the grown MiCoMo communities, indicating the transition of microbial communities in MiCoMo, yet the cultured samples were non-overlapping and could be easily traced back to their corresponding original donor.

This was confirmed with a PERMANOVA analysis (Supplementary Table 1). Between each pair of communities from independent volunteers, PERMANOVA resulted in a pseudo-F test score of >30 and a p-value of less than 0.001 after 999 permutations. When comparing between the mixed community obtained from a mixed inoculum (individuals A + C) to the respective inocula, the pseudo-F score was lower, especially for individual C, which was in line with the PCoA plots.

**Figure 6.**
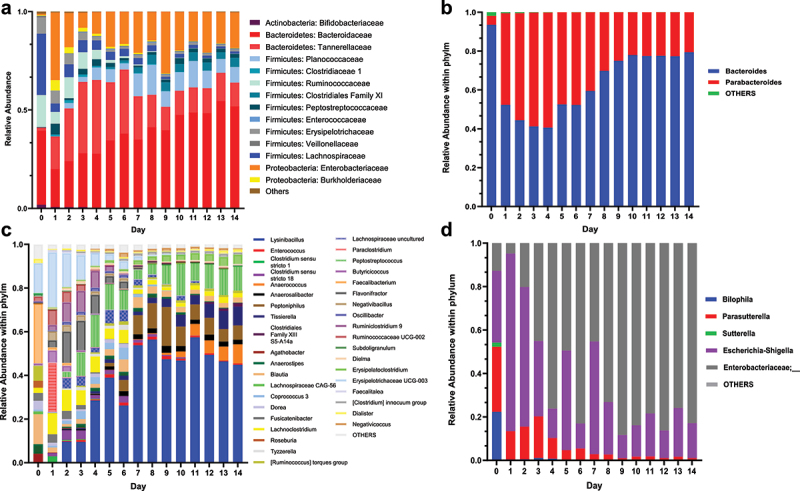
Representative dynamics of bacterial taxonomy in MiCoMo over 14 d culture for Individual A. Day 0 indicates original fecal sample, Day 1–14 indicate cultured samples from MiCoMo, averaged over 3 technical replicates. ASVs that didn’t account for at least 1% of total abundance in any day were grouped in Others. A) Family-level taxonomy of the whole community B) Genus-level taxonomy within the Bacteroides phylum C) Genus-level taxonomy within the Firmicutes phylum D) Genus-level taxonomy within the Proteobacteria phylum.

Last, we assigned taxonomy to MiCoMo-developed communities to explore the compositional dynamics over 14 d. We analyzed the overall community taxonomy at the family level and investigated the composition of individual genera within each phylum (Figure 6 and Supplementary Figure S1 – S3).

As detailed above, one important feature of MiCoMo is its ability to develop individual-specific microbial communities, leading to individual-specific temporal dynamics. Nevertheless, some general trends could be identified among the commonly found gut microbiota phyla. In all individuals, we observed an expansion of Bacteroidetes from 0.31–37.6% in the inocula to 30.0–67.2% in stabilized complex cultures. This expansion was largely contributed by *Bacteroidaceae* and *Tannerellaceae* families, most notably bacteria from the *Bacteroides* and *Parabacteroides* genera, both common members of the human gut microbiota.^22,23^ This increase was accompanied by an overall loss of Firmicutes, especially those of Clostridiales order, which exhibited ~5-fold decrease in abundance for multiple volunteers. These decreases were most significant among the families of *Lachnospiraceae* and *Ruminococcaceae*. Notably, *Faecalibacterium prausnitzii*, a common commensal gut microbiota,^24^ which consisted of 15–30% of abundance in the original fecal samples in this study, did not manage to maintain a niche in MiCoMo.

The phylum of Actinobacteria, although a common member of human gut microbiota, is usually not found in high prevalence.^25^ Indeed, for most volunteers, we found Actinobacteria consisting less than 2% of ASVs, with the exception of individual B with 20% of ASVs assigned to Actinobacteria, the majority of which belong to the *Bifidobacteriaceae* family. This family was generally not supported by MiCoMo, and gradually decreased in abundance over the first few days of culture.

We did not observe any sustained expansion of facultative aerobic bacterial taxa belonging to the Proteobacteria phylum in MiCoMo, which remained less than 10% of the overall community; except for individual A, where Proteobacteria consisted of ~20% of community once stabilized. Indeed, we observed a short expansion of Proteobacteria during the first few days of culture, whereby they could occasionally make up as much as 20–25% of the ASVs. Such expansion was however typically suppressed after 3–4 d of culture, and the relative abundances of Proteobacteria were maintained at low levels after this stabilization period.

## Discussion

Given the increased attention to the vital role the gut microbiota plays in human health, *in vitro* systems for controlled experimental investigation have been extensively developed and implemented. Most notable among them are bioreactors for propagating and maintaining microbial communities derived directly from human fecal samples. However, despite their versatility and functionalities, one common limitation is that these systems are typically dependent on additional specialized expensive lab equipment and setup, such as anaerobic chambers or multi-channel pumps.^15^ Despite the commercialization of several models, most systems’ applications are limited within the lab of creation and the accessibility world-wide is usually restrained.^26^ As such, one of our primary foci for MiCoMo design was to ensure the system is low-cost and can be easily established by most labs. The whole system costs ~$1,500 CAD and can be assembled by personnel with limited engineering experience with ease (A list of components and price can be found in Supplementary Information). The small working volume of reactors (30 ml) and the compact design also reduce the system footprint, allowing the whole MiCoMo to fit on a typical lab bench or within a biosafety cabinet. Compared to other small scale-systems, MiCoMo operates independently of anaerobic chambers, which are usually expensive and cumbersome to setup and maintain. Notably, MiCoMo is also equipped with pH control, which also makes it suitable for mimicking physiological conditions leading to a pH shift in the GI tract. Together, these features allow for easy replication across laboratories, as well as multiplexing capabilities by establishing multiple sets of MiCoMo systems in parallel within relatively small spaces, if desired.

Our validation experiments demonstrated that MiCoMo can maintain anoxic conditions at specific pH levels, leading to suitable growth conditions for two strict anaerobes and allowing for investigators to adjust the pH according to their own experimental needs. At a more fundamental level, the triplicate reactors of MiCoMo can be easily reconfigured to connect to each other in series instead of in parallel. Individually equipped with pH control system, these reactors could, when connected in series, mimic the human GI tract from stomach to colon by adjusting the pH setpoint and inoculating with different samples. This setup would enable the investigation of how the gut microbiota responds to perturbations along the GI tract.

When analyzing complex microbial community stability and structure, we were most interested in whether our system achieved a performance comparable to the currently available *in vitro* systems and animal models. Our observed Shannon index from fecal samples is comparable to previously published values that typically range from 4 to 6.5 for healthy individuals.^27^ The decrease in alpha diversity and in observed ASVs for microbial communities grown in MiCoMo likely reflects a selection process by the specific growth conditions used (media, retention time, etc.) as well as the initial composition of the fecal inoculum. Notably, due to lack of incubation time (feed cycles were immediately started after inoculation), some slow-growing bacteria might have been washed off during the initial transition period before being able to adapt to the new *ex vivo* conditions. Such selection processes were commonly observed in other *in vitro* system as well.^15,28^ Importantly, despite the decrease, MiCoMo-grown communities demonstrated an alpha diversity similar to that observed in previously reported *in vitro* systems after stabilization,^15,27^ indicating that MiCoMO was able to support growth of complex and diverse communities from a variety of fecal samples.

A big challenge for assessing stability of microbial communities in *in vitro* systems lies in the lack of a clear consensus for defining community stability and distinguishing natural variations within communities from major community shifts. Here, by adopting previously published analyzes and diversity metrics, we are able to directly compare our system to a previously validated *in vitro* system, the MBRA.^15^ Notably, Auchtung et al. not only reported stability metrics of their *in vitro* system, but also analyzed and compared these metrics to those observed in mouse models.^29^ It was reported that the six weaned mice with stable microbial communities analyzed by Auchtung et al. demonstrate a day-to-day variation in Bray-Curtis similarity (daily similarity) of 0.79 ± 0.06, and a between-replicate similarity of 0.71 ± 0.05. Meanwhile, the MBRA system had a daily similarity of 0.74 ± 0.05 and a between-replicate similarity of 0.54 ± 0.07 to 0.61 ± 0.08 during stable operations, depending on the volunteer. The MiCoMo system, with a daily similarity of 0.81 ± 0.07 during stable operation (all volunteers included; Day 5 – Day 14 for individual B and Day 3 – Day 14 for all other individuals) and a between-replicate similarity of 0.72 **±** 0.13, thus exhibited similar performance (no significance difference between MiCoMo and mice, unpaired t-test with p > .5 for both categories). This demonstrates that MiCoMo is able to support stable microbial community growth, with variations comparable with an *in vivo* mouse model and a previously reported *in vitro* systems, from various fecal inocula, after a timeframe of 3–5 d. This stabilization time required is shorter than the 7-d-period reported for MBRA,^15^ and to that required by other existing commercial systems such as the SHIME (2 weeks).^10^

When analyzing the principal component analysis plots, we observed that the communities developed from the pooled sample (Individuals A and C) interestingly clustered closely and almost exclusively with one of its source donors, individual C, by Jaccard distance for all three replicates, whereas this was not the case for the Bray-Curtis distance. The difference between replicate reactors from the same pooled fecal sample emphasizes the need for technical replicates. Further, this distinction hints at the importance of using the number of individual human donor samples as the statistical inference unit when conducting large-scale perturbation analysis, as suggested by Walter et al.,^8^ as opposed to only using the number of technical replicates (replicate mice or bioreactors with same inoculum).

Looking at the taxonomy of MiCoMo-grown microbial communities, except for a selected few known members of the gut microbiota, we limited the taxonomic assignment to the genus level, as there is extensive literature discussing the limitations of 16S rRNA sequencing with selected variable regions to reach species-level identification.^30,31^

We first report an overall decrease in the relative abundance of Firmicutes, likely due to their extreme intolerance to oxygen (loss of cultivability after <2 min of oxygen exposure for some species has been reported^32^), in addition to possible nutrient preferences. Other validated *in vitro* systems have reported similar observations, with either a decrease in abundance or a complete loss of members of this phylum.^15,28^ In addition, the expansion of facultative bacterial species belonging to the Proteobacteria phylum has been observed in various *in vitro* fermentation systems,^15,28^ likely due to their high resilience to oxygen exposure and short doubling time.^33–35^ Interestingly, we did not observe this phenomenon for most volunteers in MiCoMo after the first few days of inoculation. Rather, we observed an expansion of several known members of the gut microbiota, such as *Bacteroides uniformis* and *B. thetaiotaomicron*, both species being strict gut anaerobes.^22,36,37^ These observations indicate that some of the underlying microbial interactions known to take place in the gut could also be occurring in MiCoMo, such as limiting the expansion of Proteobacteria. Importantly, MiCoMo does not seem to select for the most adaptable and aero-tolerant species, although these may establish their niche early on during the stabilization period.

In this paper, we demonstrate that MiCoMo is able to support stable and distinct microbial communities from different volunteers, using a previously validated culture medium, as a first proof-of-functionality of MiCoMo. However, the strength of the MiCoMo system lies in its versatility: with user-customizable pH setpoint, gas sparging and feed schedules, one can easily adjust the MiCoMo environment to better accommodate individual-specific gut conditions. For instance, the pH setpoint can be decreased along with a gas sparging with increased interval in order to mimic the gut environment of IBD patients with reduced pH and increased oxygen concentration.^38,39^ In order to better support a mucosal microbial communities, mucin could also be supplemented into the system, as previously done in the SHIME system.^40^

## Methods

### Media Preparation

Modified Gifu Anaerobic Medium (mGAM) (05433, Hyserve) was chosen as the media for MiCoMo according to previously published studies.^41^ The medium was prepared by dissolving 41.7 g powder in 1 L distilled water and sterilized by autoclaving at 121°C for 30 min. 0.01% Antifoam 204 (A6426, Sigma)^42^ was added to the media to minimize foam formation during the experiment.

### Validation with growth of strict anaerobes

For inoculating strict anaerobes, mGAM was pre-reduced in an anaerobic chamber (COY laboratory, functioning with 5%H_2_, 20% CO_2_, and balance N_2_) 24 h before usage. *Clostridium beijerinckii* (ATCC 51743) and *Bacteroides fragilis* (32-6-I 11 MRS AN) were each seeded in 5 mL pre-reduced media and left overnight at 37°C in an anaerobic chamber. 3 ml of overnight culture of each bacterium was then inoculated in individual MiCoMo reactors with 27 mL media supplemented with 0.4 g/L L-cysteine. In addition, 1 ml of overnight culture was serially diluted and seeded on pre-reduced mGAM agar plates. Colony counting was performed 48 h after incubation of agar plates in an anaerobic chamber at 37°C. OD600 vs. CFU·mL^−1^ curves were generated for each bacterial isolate by measuring the OD of samples of known concentrations obtained from colony counting. The final seeding density in MiCoMo was 1.76 × 10^8^ CFU·mL^−1^ for *B. fragilis* and 1.10 × 10^6^ CFU·mL^−1^ for *C. beijerinckii*. One sample was taken immediately after inoculation for OD600 measurement at time 0 (T0). At specific time points, output pumps were manually turned on to collect 0.1 mL of sample, and the OD600 of samples were measured with a ND-1000 Nanodrop spectrophotometer (Thermo Scientific). The collection schedule was every hour until T7 for *B. fragilis* and every 2 hours until T8 for *C. beijerinckii*, then at T24 and T48 for both isolates. In these experiments, the MiCoMo automatic pH and fluidic adjustments were disabled, and 3 ml of fresh media were added every 24 h to compensate for evaporation.

### Fecal sample collection and preparation

This study was conducted following McGill University’s approved ethics protocol A04M2715B. Fecal samples from four anonymized healthy unrelated volunteers without history of antibiotic usage within 6 months prior to participation were collected, weighed, and aliquoted in sterile 50 mL Falcon tubes within 15 minutes of collection. The aliquoted samples were then stored in −80°C until use.

Prior to inoculation, fecal samples were resuspended in phosphate buffered saline pre-reduced with 4 g/L L-cysteine at 20% w/v concentration (rPBS, with pH = 7). The fecal slurry was centrifuged at 200 g for 3 minutes to remove large cellular debris. 3 ml of supernatant were then inoculated in individual MiCoMo reactors with 27 mL L-cysteine-supplemented media (see below), resulting in a final fecal sample concentration of 2% w/v, for each sample.

### MiCoMo operation and sampling

The day prior to fecal inoculation, MiCoMo reactors were sterilized by incubating in 70% ethanol for 1 h. The system was then assembled in a biosafety cabinet and 10% bleach was run through all the piping connections and reactors for 30 min. Sterile MilliQ water was then flushed in the system for 5 min. pH probes were sterilized by soaking in 10% bleach for 1 h, followed by rinsing with sterile MilliQ water.

Upon fecal inoculation, 1 mL of 40 g·L^−1^ L-cysteine (C1276-50 G, SIGMA) solution was added to 99 ml of sterile mGAM with antifoam to enhance the oxygen scavenging and establishment of anoxia. This additional L-cysteine supplementation was only added to media for initial inoculum and not applied to media feed during the rest of experiment. The supplemented mGAM medium was then incubated in a water bath at 100°C for 30 min to remove dissolved oxygen, and the container overhead was flushed with sterile nitrogen gas before being left to cool down to room temperature. Anoxic media was then added into individual reactors in a water bath maintained at 37°C. The nitrogen flushing and pH control system were subsequently initiated. The system was adjusted to a pH setpoint and maintained for at least 30 min prior to the inoculation with fecal matter via the seeding port of the reactors. For all experiments with fecal samples, the pH was maintained at 6.7 with ± 0.1 tolerance.

For practical experimental purposes, we started 4-hour feed cycles immediately after inoculation. At the end of each cycle, 4 mL (13%) of reactor content was removed from each reactor and 4.5 ml of fresh media was added. The excess media was necessary to compensate for liquid loss due to evaporation (which resulted in ~3 ml loss per day). Once every 24 h, 4 mL of removed content (one cycle) was collected for each reactor and immediately centrifuged at 14,000 g to precipitate the bacteria. The supernatant and pellets were then stored at −80°C. In each experiment, the feed cycles were maintained for 14 d.

### DNA extraction and sequencing

DNA in fecal samples for each individual were extracted from prepared inoculum of MiCoMo (supernatant of fecal slurry) with both QIAamp PowerFecal Pro DNA Kit (51804, Qiagen, Germany) and DNeasy UltraClean Microbial Kit (12224–50, Qiagen, Germany). DNA from the daily reactor samples were extracted with DNeasy UltraClean Microbial Kit only. DNA extraction and purification followed the supplier’s protocols. DNA amplification and amplicon library preparation/sequencing were performed by the UQAM genomics platform (CERMO-FC genomic platform, Department of biological sciences, Université du Québec à Montréal). Briefly, extracted DNA samples were amplified with primer pairs specific to the V4-V5 region of bacterial 16S rRNA (515 F/926 R) and sequenced with Miseq V3 kit. The forward and reverse primer sequences were: 5’ – GTGYCAGCMGCCGCGGTAA – 3’ and 5’ – CCGYCAATTYMTTTRAGTTT – 3’, respectively.^43^ Sequencing reactions were performed on a Miseq using MiSeq reagent kit v3 (600-cycles; Illumina). The reads were 2 × 300 bp with an average depth of ~30,000 reads per sample.^41^ Adapters were trimmed after sequencing and raw reads were demultiplexed with Local Run Manager.

### Sequencing data analysis

FASTQ data of paired end sequences were denoised using the DADA2 plugin of QIIME2, version 2020.11.^44^ All sequences were trimmed at 25 bp for both forward and reverse reads to remove primer pairs and further truncated at 260 bp for forward reads and 230 bp for reverse reads. All other settings of DADA2 remained as default. Amplicon sequencing variants (ASVs) were further analyzed with *diversity* plugins of QIIME2 with a sampling depth of 4,100 to include all samples while ensuring taxonomic recovery and recapturing sample diversity patterns.^45^ Taxonomy was assigned to the ASVs by a Naïve-Bayes classifier pre-trained with SILVA rRNA database^46^ using only regions specified by the primer used in these experiments. Alpha and beta diversity analyses and PERMANOVA analyzes were done with the *diversity* plugin of QIIME 2. The Bray-Curtis similarity (1 – Bray-Curtis distance) was applied as beta diversity metrics basing on a previously published approach^47^ for examining the stability of microbial cultures.^15^ Daily rate of change of Bray-Curtis similarity was computed by using a 3-point moving window slope. Graphs were plotted with either QIIME 2 *emperor* plugin or GraphPad Prism 9. Statistical tests were conducted with GraphPad Prism 9.

## Conclusions

In conclusion, we developed MiCoMo, an optimized, fully controllable miniaturized, pH-controlled, and anerobic bioreactor system for simulating the gut environment. We showed that it allows for fast stabilization of complex microbial communities (approx. 3–5 days), while sustaining microbial diversity from individual volunteers. We believe its small footprint, low cost, and ease of fabrication will allow for easy replication across labs studying the effect of various perturbations on individual gut microbial communities with high throughput. We expect future developments of MiCoMo to focus on two aspects: further miniaturization and increased multiplexing capacity along with compartmentalization of the human GI tract. The size of the pH probes currently dictates the size of the bioreactors, and can be replaced by miniaturized pH probes (more expensive) or probes with minimal footprint and low cost.^48^ Then, due to its inherent modular nature, MiCoMo can be modified to integrate the biologic compartmentalization of the human GI tract by introducing a small intestine chamber inoculated with small intestine microbiota samples, and/or a stomach chamber for mimicking food digestion. These modifications are also compatible with increased multiplexing capacity by duplicating existing modules without modifying the designs. Finally, future development might make it possible to optionally incorporate host cells into MiCoMo, thereby incorporating back host–microbiota interactions while controlling environmental parameters.

## Data Availability

16S rRNA gene sequencing data can be accessed in the SRA database under accession number PRJNA819079 (https://www.ncbi.nlm.nih.gov/bioproject/PRJNA819079). Code related to the analysis and operation of MiCoMo has been deposited in GitHub (https://github.com/focussash/MiCoMo-Manuscript).
